# The humanistic burden of advanced non-small cell lung cancer (NSCLC) in Europe: a real-world survey linking patient clinical factors to patient and caregiver burden

**DOI:** 10.1007/s11136-019-02152-6

**Published:** 2019-03-02

**Authors:** Robert Wood, Gavin Taylor-Stokes, Fiona Smith, Carlos Chaib

**Affiliations:** 1 Adelphi Real World, Adelphi Mill, Grimshaw Lane, Bollington, Macclesfield, SK10 5JB UK; 2 Bristol-Myers Squibb, Madrid, Spain

**Keywords:** Caregiver burden, Physical function, Lung cancer, Quality of life, Work productivity

## Abstract

**Purpose:**

Advanced non-small cell lung cancer (aNSCLC) impacts the lives of patients and their caregivers. This analysis examined the association between patient clinical characteristics and patient and caregiver humanistic burden.

**Methods:**

Data for patients with aNSCLC and their informal caregivers in France, Germany and Italy, were collected between May 2015 and June 2016 via chart review and patient and caregiver surveys. Patients and caregivers completed validated instruments to evaluate their health state (EuroQol-5-dimensions-3-levels [EQ-5D-3L]), work and activity impairment (Work Productivity Activity Impairment [WPAI]) and health-related quality of life (HRQoL; European Organisation for Research and treatment of Cancer Quality of Life Questionnaire [EORTC QLQ-C30]). Caregivers also completed the Zarit Burden Interview (ZBI). Univariate and regression analyses were stratified by patient Eastern Cooperative Group Performance Status (ECOG-PS 0, 1, 2 or 3/4).

**Results:**

In total, 1030 patients and 427 accompanying informal caregivers participated. Regression analyses indicated that patients reported lower EQ-5D-3L utility index, EQ-VAS and EORTC QLQ-C30 global health status and greater work and activity impairment with worsening ECOG-PS (all *p* < 0.05). Caregivers also reported greater activity impairment and higher ZBI scores with worsening ECOG-PS of the patient they were providing care for (all *p* < 0.05).

**Conclusions:**

As patients’ functionality deteriorates as measured by the ECOG-PS, so do their outcomes related to health utility, work productivity, activity impairment and HRQoL. This deterioration is also reflected in increased caregiver burden and activity impairment. There is a need for interventions to maintain patients’ physical function to relieve the humanistic burden of both patients and caregivers.

**Electronic supplementary material:**

The online version of this article (10.1007/s11136-019-02152-6) contains supplementary material, which is available to authorized users.

## Introduction

Non-small cell lung cancer (NSCLC) accounts for approximately 85% of all lung cancer diagnoses in Europe [1]. The majority of patients are not diagnosed until their disease has reached an advanced stage at which time it is associated with a poor prognosis, even with current treatment options [2]. Moreover, around half of those initially diagnosed with early stage disease (stage I or II) will eventually advance to metastatic NSCLC. Advanced NSCLC (aNSCLC; defined as stage IIIB or IV disease) and its current treatments (including systemic chemotherapy, immunotherapy and targeted agents) impose a significant detrimental impact on the lives of patients [3, 4] and their family and friends [5], especially those providing informal care for a family member with aNSCLC. Patients and their informal caregivers (family members or friends) face physical, emotional and financial challenges that have the potential to significantly impact on their health-related quality of life (HRQoL) and psychological health [6–9].

Patients with aNSCLC have been shown to have worse HRQoL not only when compared with the general population but also when compared with patients suffering from other advanced cancer types [10]. In 2015, a survey of 163 family caregivers for patients with lung cancer (> 75% of whom had stage III or IV disease) found that caregiver distress levels increased as patient quality of life (QoL) declined [11]. A recent prospective cross-sectional study of 91 patient-caregiver dyads found that patient HRQoL was a more relevant driver of caregiver burden (poorer HRQoL associated with greater caregiver burden) than disease stage [12]. Limited information exists on the clinical characteristics that contribute to the humanistic burden incurred by patients with aNSCLC and their caregivers, i.e. the impact aNSCLC has on a patient’s/caregiver’s physical, social, emotional and/or financial well-being. In an evaluation of 43 patient-caregiver dyads, lung cancer symptoms and the presence of anxiety/depression in patients were shown to be positively related to caregiver burden and anxiety/depression [13]. This small study highlights the close association between patient and caregiver outcomes. Interdependence of anxiety and depression has also been reported for patient–caregiver dyads of patients with newly diagnosed incurable cancers [14].

Further insights into the clinical characteristics that drive the humanistic burden patients and their caregivers experience will provide useful guidance for health care practitioners and service providers. Such insights will enable physicians and policy makers to provide focused care and service provision with the aim of improving the QoL for patients with aNSCLC and their caregivers. For further understanding in this area, the current research was undertaken to examine the association between patient clinical characteristics including functional status as measured using the Eastern Cooperative Oncology Group—Performance Status (ECOG-PS), and patient and caregiver humanistic burden with a view to identifying modifiable factors that might be targeted to mitigate this burden for both patients and their caregivers.

## Methods

The objective of the study was to examine the association between patient functional status (as measured using the ECOG-PS) and the humanistic burden of patients with aNSCLC and their caregivers.

Data were derived from a real-world, multi-centre, point-in-time study of patients with stage IIIB or IV NSCLC and their caregivers conducted in France, Germany and Italy. The point-in-time design permitted collection of retrospective data from individual patient clinical records. Data collection took place between May 2015 and June 2016 and consisted of a medical chart review, undertaken by the treating physician, a patient survey and a caregiver survey. All data were fully de-identified, collated, aggregated and coded to permit linkage between physician-reported data, patient responses and their caregiver responses. The study protocol was approved by a centralized Institutional Review Board (Freiburg Ethics Commission International).

Participating physicians invited patients with aNSCLC attending their clinic and their accompanying informal caregivers to participate in the study. A combined information sheet and informed consent form fronted both the patient and caregiver paper questionnaires. Patients and caregivers received an information sheet outlining: the objectives of the study, that completion of the questionnaire was entirely voluntary, that they were free to withdraw at any point and assurances that any responses they gave would remain confidential. Patient and caregiver informed consent was confirmed by an anonymized tick box on the front page of the paper questionnaire distributed by the consulting physician. Patients and/or caregivers, who did not wish to participate, did not complete a questionnaire. Participation was voluntary, and both patients and caregivers were free to withdraw at any time without giving a reason.

### Patient population

To be eligible to participate in the study, patients (male or female; ≥ 18 years) were required to have histologically or cytologically confirmed stage IIIB or IV NSCLC and to have initiated their first therapy for the treatment of aNSCLC at least 1 calendar month prior to data collection. All patients meeting these criteria, and who were willing and able to complete the patient survey were eligible for participation. As the aim of this research was to examine a real-world cohort of patients, those enrolled in clinical trials at the time of the survey were not eligible to participate.

### Caregiver population

Primary caregivers (spouse, partner, child, other relative or friend) who self-identified as the individual providing all or the majority of the informal (unpaid) care for a patient with stage IIIB or IV NSCLC who were ≥ 18 years and were willing and able to complete a caregiver survey were eligible to participate in the study. Those offering formal, paid caregiving, or volunteer caregiving were not eligible to participate.

### Data collection

The medical records of each participating patient were reviewed, and data were extracted by the treating physician. Data were captured in an electronic patient record form and included, but were not limited to, patient demographics (age, gender) and clinical characteristics (ECOG-PS, disease stage, histology, disease history, comorbidities and treatment history).

Additionally, patients were asked to complete a short paper-based survey. As part of the survey, patients completed a number of specific validated instruments to evaluate their health state, work productivity, activity impairment and HRQoL. All questionnaires were delivered in the validated local language format (French, German or Italian). Patients’ HRQoL was measured using the EuroQol five-dimensional questionnaire three level version (EQ-5D-3L) [15]. The EQ-5D is a non-disease-specific tool validated to evaluate five dimensions of health: mobility, self-care, usual activities, pain/discomfort and anxiety/depression. The three-level (3L) version assesses each item as ‘having no problems’, ‘some or moderate problems’ or ‘unable to do/having extreme problems’. In terms of interpretation, a higher EQ-5D-3L score indicates a better health state. The questionnaire also includes a visual analogue scale (VAS) through which the respondents rate their own perceived health status from ‘best imaginable health state’ to ‘worst imaginable health state’. Work productivity and activity impairment was measured using the Work Productivity and Activity Impairment: General Health (WPAI:GH) questionnaire [16]. The WPAI-GH is a validated, non-disease-specific tool and consists of six items covering employment status, hours missed from work due to health problems, hours missed from work due to other reasons, hours actually worked, how any health problems have affected productivity at work and how any health problems have affected regular daily activities. These six questions are used to derive four domains, work time missed (absenteeism), impairment while working (presenteeism), overall work impairment and activity impairment. Patient HRQoL was further measured using the non-disease-specific tool EORTC QoL Questionnaire (EORTC QLQ C30) [17], a questionnaire specifically designed and validated to measure the QoL of patients with cancer. The EORTC QLQ-C30 is a 30 item self-completed questionnaire yielding 5 functional scales (physical, role, emotional, social and cognitive), 3 symptom scales (fatigue, nausea and vomiting and pain), a global health status/QoL scale and 6 single items (dyspnoea, insomnia, appetite loss, constipation, diarrhoea and financial difficulties). Higher scores for the global health status and functional scales indicate a higher QoL and level of functioning, respectively, while higher scores for the symptom scales/items indicate higher level of symptomatology.

Accompanying caregivers providing informal care who agreed to take part in the study were invited to complete a short paper-based survey, which included the EQ-5D-3L, WPAI-GH and the Zarit Burden Interview (ZBI) [18]. The ZBI is a validated 22-item scale used to measure feelings of burden among caregivers for patients with a range of medical and psychological conditions. Higher ZBI scores are indicative of higher levels of burden, while scores ≥ 24 are considered to indicate the respondent to be at risk of depression [19]. Initially developed for the evaluation of caregivers of patients with dementia, the ZBI has been validated for the evaluation of caregivers of patients with cancer [20].

### Statistical analyses

Descriptive statistics are presented throughout. Analyses were stratified by ECOG-PS of the patient and by the presence of comorbid emphysema and anxiety/depression. These comorbidities and others collected in the physician review were selected based on their association with overall survival and quality of life, in patients with lung cancer [21, 22]. Statistical significance was assessed using Mann–Whitney U (2 subgroups) and Kruskal–Wallis (3 + subgroups) tests for numeric and ordinal outcomes, and Fisher’s Exact (2 subgroups) or Chi-squared (3 + subgroups) tests for nominal outcomes. Regression analyses were also undertaken to explore the impact of the patient’s functional status (ECOG-PS) on patient and caregiver outcomes, while adjusting for basic demographics (age, sex, country of origin, smoking status; caregiver age and sex also included in caregiver outcome models) and clinical characteristics (disease stage and duration, prior chemotherapy, presence of brain metastases). All analysis was performed using Stata 15 software [23].

## Results

Overall, 1030 consulting patients and 427 accompanying informal caregivers were recruited to the study via 141 treating physicians.

Patient mean age was 64.5 years (standard deviation [SD] 10.1), 65.9% were male, and 77.9% were either current or ex-smokers (Table 1). The majority (88.4%) had stage IV NSCLC at the time of study completion and non-squamous histology (70.3%). The mean duration since the diagnosis of NSCLC was 35.9 weeks. Over two-thirds (70.5%) of patients were receiving first line therapy with 29.5% receiving second or later lines of therapy. Most were receiving platinum-based doublet chemotherapy (44.3%), single-agent chemotherapy (20.4%) or an epidermal growth factor receptor (EGFR) inhibitor (15.0%). Of the 302 patients receiving a second or later line therapy, 15.6% and 11.9% were receiving an EGFR inhibitor and/or a Programmed death-1 inhibitor, respectively. Approximately one-fifth (19.4%) of patients had an ECOG-PS of 0, 43.5% 1, 27.4%, 2 and 9.7% an ECOG-PS of 3 or 4, while 10.4% and 20.7% of patients were diagnosed with comorbid emphysema and/or anxiety/depression, respectively.

**Table 1:** Patient demographics and clinical characteristics

Characteristic	Overall *N* = 1030	Current ECOG performance status			
		0 *n* = 200	1 *n* = 448	2 *n* = 282	3 or 4 *n* = 100
Age, mean (SD) years	*n* = 1028	*n* = 200	*n* = 447	*n* = 281	*n* = 100
	64.5 (10.1)	59.2 (9.2)	63.5 (9.8)	67.7 (8.9)	70.5 (10.2)
Male, *n* (%)	*n* = 1030	*n* = 200	*n* = 448	*n* = 282	*n* = 100
	679 (65.9)	123 (61.5)	308 (68.8)	182 (64.5)	66 (66.0)
Smoking status, *n* (%)	*n* = 1010	*n* = 197	*n* = 439	*n* = 277	*n* = 97
Never-smoker	223 (22.1)	69 (35.0)	88 (20.0)	47 (17.0)	19 (19.6)
Current/ex-smoker	787 (77.9)	128 (65.0)	351 (80.0)	230 (83.0)	78 (80.4)
Disease duration, mean (SD) weeks	*n* = 1015	*n* = 200	*n* = 436	*n* = 280	*n* = 99
	35.9 (44.9)	29.8 (45.0)	33.7 (38.5)	43.6 (51.7)	35.9 (47.9)
Histological type, *n* (%)	*n* = 1030	*n* = 200	*n* = 448	*n* = 282	*n* = 100
Non-squamous	724 (70.3)	148 (74.0)	313 (69.9)	190 (67.4)	73 (73.0)
Squamous	306 (29.7)	52 (26.0)	135 (30.1)	92 (32.6)	27 (27.0)
Current NSCLC stage, *n* (%)	*n* = 1030	*n* = 200	*n* = 448	*n* = 282	*n* = 100
Stage IIIb	119 (11.6)	24 (12.0)	53 (11.8)	29 (10.3)	13 (13.0)
Stage IV	911 (88.4)	176 (88.0)	395 (88.2)	253 (89.7)	87 (87.0)
Line of therapy, *n* (%)	*n* = 1022	*n* = 199	*n* = 441	*n* = 282	*n* = 100
First	720 (70.5)	173 (86.9)	315 (71.4)	160 (56.7)	72 (72.0)
Second or later	302 (29.5)	26 (13.1)	126 (28.6)	122 (43.3)	28 (28.0)

*ECOG* Eastern Cooperative Oncology Group, *NSCLC* non-small cell lung cancer, *SD* standard deviation

The mean age of caregivers was 53.5 years (SD 12.5), 72.6% were female, the majority were either the patient’s partner/spouse (54.9%) or child (31.9%) and over half of caregivers (56.9%) reported that the patient received no additional formal or informal support (Table 2).

**Table 2:** Caregiver demographics

Characteristic	Overall *N* = 427	Current ECOG performance status			
		0 *n* = 66	1 *n* = 181	0 *n* = 129	3 or 4 *n* = 51
Age, mean (SD) years	*n* = 425	*n* = 66	*n* = 179	*n* = 129	*n* = 51
	53.5 (12.5)	52.8 (11.6)	52.7 (12.0)	53.1 (13.3)	58.2 (12.4)
Female, *n* (%)	*n* = 423	*n* = 66	*n* = 178	*n* = 129	*n* = 50
	307 (72.6)	47 (71.2)	131 (73.6)	93 (72.1)	36 (72.0)
Relationship to patient, *n* (%)	*n* = 426	*n* = 66	*n* = 181	*n* = 128	*n* = 51
Partner/spouse	234 (54.9)	47 (71.2)	100 (55.2)	61 (47.7)	26 (51.0)
Mother/father	3 (0.7)	0 (0)	1 (0.6)	0 (0)	2 (3.9)
Friend/neighbour	13 (3.1)	3 (4.5)	5 (2.8)	4 (3.1)	1 (2.0)
Daughter/son	136 (31.9)	13 (19.7)	61 (33.7)	49 (38.3)	13 (25.5)
Sister/brother	11 (2.6)	2 (3.0)	4 (2.2)	3 (2.3)	2 (3.9)
Other family member	12 (2.8)	1 (1.5)	4 (2.2)	6 (4.7)	1 (2.0)
Other	3 (0.7)	0 (0)	0 (0)	1 (0.8)	2 (3.9)
None	14 (3.3)	0 (0)	6 (3.3)	4 (3.1)	4 (7.8)
Additional support provided to the patient, *n* (%)	*n* = 406	*n* = 61	*n* = 169	*n* = 125	*n* = 51
None	231 (56.9)	42 (68.9)	103 (60.9)	63 (50.4)	23 (45.1)
Formal help only	38 (9.4)	1 (1.6)	8 (4.7)	15 (12.0)	14 (27.5)
Formal + informal help	61 (15.0)	9 (14.8)	32 (18.9)	16 (12.8)	4 (7.8)
Informal help only	76 (18.7)	9 (14.8)	26 (15.4)	31 (24.8)	10 (19.6)

*ECOG* Eastern Cooperative Oncology Group, *SD* standard deviation

### Humanistic burden on patients

**Fig. 1:**
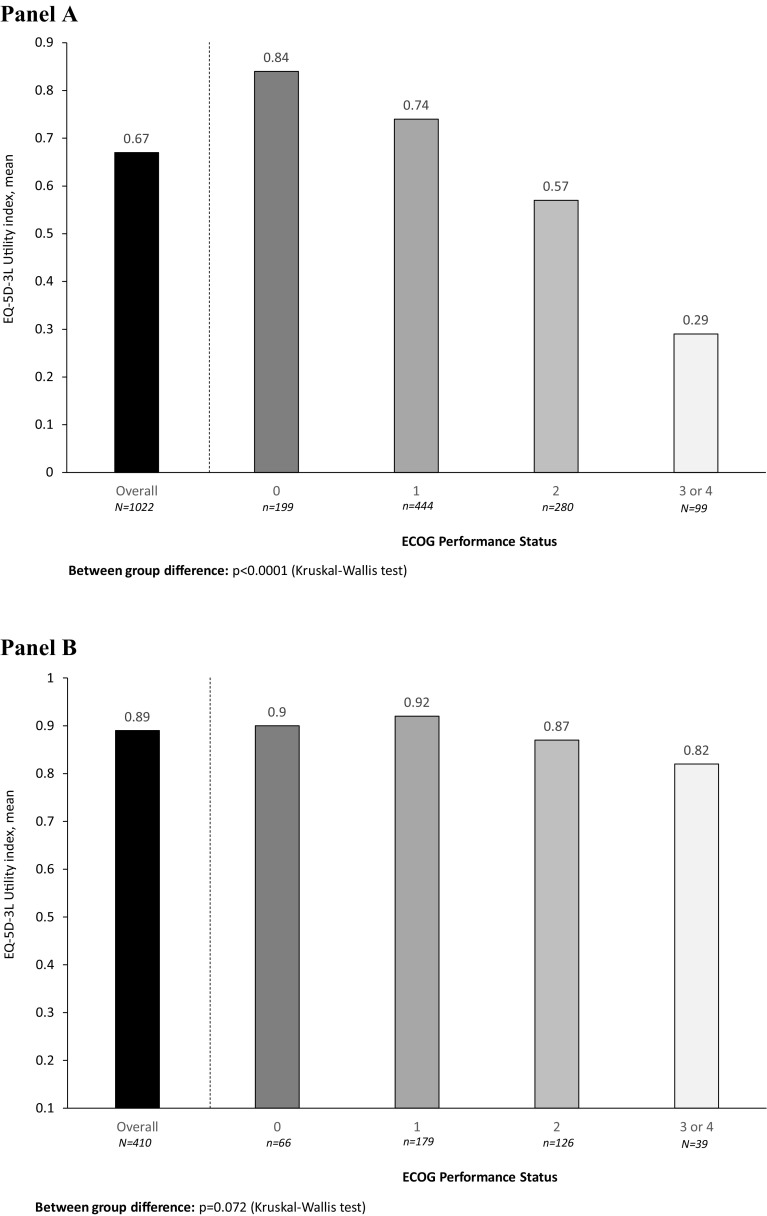
Patient (Panel **A**) and caregiver (Panel **B**) EQ-5D-3L stratified by patient ECOG Performance status

The mean EQ-5D-3L score for the 1030 patients participating in the study was 0.67 (SD 0.31) (Fig. 1). Some or extreme problems were reported by at least 40% of patients across all five domains (mobility, 53.8%; self-care, 40.7%; usual activities, 59.6%; pain/discomfort, 71.9%; anxiety/depression, 64.2%). Patients reported a mean EQ-5D VAS score of 57.4 (SD 18.1).

Statistically significant (*p* < 0.0001) differences in the distribution of patient EQ-5D-3L utility indices were observed across ECOG-PS subgroups, with an apparent trend for worsening EQ-5D-3L score with declining ECOG-PS (from a mean of 0.84 for patients with an ECOG-PS of 0 to a mean of 0.29 for those with an ECOG-PS of 3 or 4; Fig. 1). Statistically significant differences in EQ-5D-3L were also observed between patients with comorbid emphysema compared to those without comorbid emphysema (means: 0.61 vs 0.67, respectively; *p* = 0.0278) and between patients with comorbid anxiety/depression compared to those patients without comorbid anxiety/depression (means: 0.55 vs 0.70, respectively; *p* < 0.0001) (Supplementary Table A).

The distributions of all fours domain of the WPAI: GH were statistically significant (all *p* < 0.05) across ECOG-PS subgroups (means: work time missed [ECOG-PS of 0—11.9%, ECOG-PS of 3 or 4—27.2%], impairment while working [24.5–60.0%, respectively], overall work impairment [28.7–81.2%, respectively] and activity impairment [37.1–73.6%, respectively]), indicating a trend for worsening impairment with declining ECOG-PS (Table 3). Whilst impairment was numerically greater for all four domains between patients with and without comorbid emphysema, only activity impairment differed significantly (means 62.6% vs 51.6%, respectively; *p* = 0.0001) (Supplementary Table A). Statistically significant differences were also observed between patients with and without comorbid anxiety/depression for impairment while working (means 41.3% vs 29.6%, respectively; *p* = 0.0101), overall work impairment (means 49.2% vs 35.2%, respectively; *p* = 0.0169) and activity impairment (means 61.0% vs 50.6%, respectively; *p* < 0.0001) (Supplementary Table A).

**Table 3:** Humanistic burden for patients overall and stratified by ECOG-PS

Characteristic	Overall (*N* = 1030)	Current ECOG performance status				*P* value
		0 *n* = 200	1 *n* = 448	2 *n* = 282	3 or 4 *n* = 100	
EQ-5D-3L utility index, mean (SD)	0.67 (0.31)	0.84 (0.20)	0.74 (0.23)	0.57 (0.31)	0.29 (0.4)	< 0.0001^a^
EQ-5D-3L						
Mobility domain, %						
No/some/extreme problems	46.2/50.2/3.6	71.5/28.0/0.5	54.4/44.5/1.1	30.1/66.0/3.9	4.0/76.0/20.0	< 0.0001^b^
Self-care, %						
No/some/extreme problems	59.3/36.2/4.5	83.4/16.6/0	70.9/27.5/1.6	41.3/54.4/4.3	10.0/63.0/27.0	< 0.0001^b^
Usual activities, %						
No/some/extreme problems	40.4/52.2/7.4	70.4/28.6/1.0	45.8/51.7/2.5	23.4/66.3/10.3	4.0/62.0/34.0	< 0.0001^b^
Pain/discomfort, %						
No/some/extreme problems	28.1/64.7/7.2	52.3/43.7/4.0	31.2/64.6/4.3	13.1/80.9/6.0	8.1/61.6/30.3	< 0.0001^b^
Anxiety/depression, %						
No/some/extreme problems	35.8/50.1/14.0	56.8/33.2/10.1	38.2/52.6/9.2	25.3/60.1/14.6	13.0/45.0/42.0	< 0.0001^b^
EQ-5D VAS, mean (SD)	57.4 (18.1)	68.1 (15.7)	60.4 (15.7)	51.6 (17.0)	39.0 (16.8)	< 0.0001^a^
Employment status, %						< 0.0001^b^
Working full time	149 (14.7)	68 (34.3)	55 (12.5)	24 (8.7)	2 (2.0)	
Working part time	65 (6.4)	15 (7.6)	40 (9.1)	7 (2.5)	3 (3.0)	
Unemployed	105 (10.3)	20 (10.1)	48 (10.9)	25 (9.0)	12 (12.0)	
Student	1 (0.1)	0 (0)	1 (0.2)	0 (0)	0 (0)	
Homemaker	82 (8.1)	20 (10.1)	36 (8.2)	18 (6.5)	8 (8.0)	
Retired	614 (60.4)	75 (37.9)	261 (59.2)	203 (73.3)	75 (75.0)	
WPAI-GH, mean (SD)						
% work time missed	15.2 (24.6)	11.9 (22.7)	14.3 (23.3)	26.6 (32.5)	27.2 (13.5)	0.0255^a^
% impairment while working	31.0 (22.4)	24.5 (20.0)	34.1 (21.5)	36.7 (25.3)	60.0 (29.4)	0.0026^a^
% overall work impairment	36.7 (25.8)	28.7 (24.2)	39.2 (23.2)	48.4 (29.1)	81.2 (9.8)	0.0005^a^
% activity impairment	52.7 (27.9)	37.1 (26.3)	49.1 (26.1)	61.9 (26.2)	73.6 (21.5)	< 0.0001^a^
EORTC QLQ-C30 domains, mean (SD)						
Global health status	48.1 (19.8)	56.8 (18.3)	50.8 (19.2)	42.5 (18.4)	34.8 (18.0)	< 0.0001^a^
Physical functioning	63.5 (25.0)	78.7 (19.3)	68.4 (21.4)	54.3 (24.2)	36.5 (21.2)	< 0.0001^a^
Role functioning	57.4 (28.5)	72.9 (25.3)	61.9 (25.7)	48.4 (27.4)	32.5 (24.4)	< 0.0001^a^
Emotional functioning	60.4 (24.6)	69.1 (25.4)	64.0 (22.0)	55.6 (23.7)	40.4 (22.9)	< 0.0001^a^
Cognitive functioning	70.6 (25.4)	80.3 (22.3)	74.1 (23.4)	64.7 (25.8)	52.0 (25.1)	< 0.0001^a^
Social functioning	64.4 (28.1)	76.3 (26.0)	68.7 (25.7)	57.0 (28.1)	42.0 (24.2)	< 0.0001^a^
Fatigue	46.3 (25.2)	30.1 (20.8)	43.6 (22.8)	53.1 (23.7)	71.1 (22.0)	< 0.0001^a^
Nausea and vomiting	24.0 (23.8)	20.8 (22.9)	22.2 (23.2)	26.3 (24.2)	32.0 (24.8)	< 0.0001^a^
Pain	36.1 (25.4)	23.0 (24.0)	33.4 (23.8)	42.0 (23.0)	58.3 (22.2)	< 0.0001^a^
Dyspnoea	38.8 (26.6)	26.6 (24.6)	35.5 (24.8)	43.2 (25.0)	65.3 (21.1)	< 0.0001^a^
Insomnia	36.4 (28.8)	26.6 (25.9)	33.8 (28.4)	41.2 (28.9)	54.3 (25.4)	< 0.0001^a^
Appetite loss	36.5 (28.9)	26.8 (26.2)	33.4 (27.5)	43.0 (30.2)	51.7 (27.0)	< 0.0001^a^
Constipation	22.2 (25.1)	17.8 (21.4)	19.7 (24.5)	24.6 (25.1)	35.3 (29.5)	< 0.0001^a^
Diarrhoea	13.5 (21.2)	13.4 (18.9)	13.3 (21.9)	13.7 (22.2)	14.1 (19.7)	0.7580^a^
Financial difficulties	22.8 (26.5)	15.4 (23.2)	18.7 (24.3)	29.0 (28.0)	38.4 (28.3)	< 0.0001^a^

*EORTC QLQ-C30* EORTC QoL Questionnaire, *EQ-5D-3L* EuroQol five-dimensional questionnaire three level version, *SD* standard deviation, *VAS* visual analogue scale, *WPAI-GH* work productivity and activity impairment: general health

^a^Kruskal–Wallis test performed

^b^Chi-squared test performed

Patients reported a mean global health status score on the EORTC-QLQ-C30 of 48.1 (SD 19.8) with the domain scores ranging from 13.5 (SD 21.2) for diarrhoea to 70.6 (SD 25.4) for cognitive functioning (Table 3). The distributions of the global health status, all functional scales, all symptom scales and all single items, with the exception of diarrhoea, differed significantly (all *p* < 0.0001) across ECOG-PS subgroups, and indicated a trend of worse QoL, functioning and symptoms for patients with declining ECOG-PS. Statistically significant differences in global health status were also detected between patients with and without comorbid emphysema (means: 43.6 vs 48.6, respectively; *p* = 0.0089), and between patients with and without comorbid anxiety/depression (means 44.8 vs 48.9, respectively; *p* = 0.0023) (Supplementary Table A).

#### Regression analyses

Regression analyses indicated that a declining functional status was associated with a worse EQ-5D-3L utility index, a worse EQ-VAS, greater activity impairment and a worse EORTC-QLQ-C30 global health status (Table 4). An ECOG-PS of 1, 2 or 3/4 was associated with a 0.05 (*p* < 0.05), 0.19 (*p* < 0.001) and 0.51 (*p* < 0.001) decrease in EQ-5D-3L utility index, respectively, compared to an ECOG-PS of 0. A similar pattern was observed for EQ-VAS (ECOG-PS 1: − 5.75, ECOG-PS 2: − 12.38 and ECOG-PS 3/4: − 24.24; all *p* < 0.001) and global health status (ECOG-PS 1: − 3.91 [*p* < 0.05], ECOG-PS 2: − 11.03 [*p* < 0.001], ECOG-PS 3/4: − 19.48 [*p* < 0.001]). An ECOG-PS of 1, 2 or 3/4 was also associated with a 7.54, 18.78 and 31.11 increase in activity impairment, respectively, compared to an ECOG-PS of 0 (all *p* <0.001). An ECOG-PS of 3/4 was associated with a 50.77 (*p* < 0.001) increase in overall work impairment, but the sample size for this regression model reduced substantially as it contained employed patients only.

**Table 4:** Regression analysis of the impact of clinical, functional and demographic characteristics of patients and outcome scores for patients and caregivers

	Patient outcomes				
	EQ-5D-3L	EQ VAS	WPAI-GH: overall work impairment	WPAI-GH: activity impairment	EORTC-QLQ-C30: global health status
N	983	970	158	944	980
Adjusted R^2^	0.3727	0.2639	0.1752	0.2148	0.1769
ECOG-PS: 1 *(Reference group* = *0)*	− 0.053* (− 0.097, − 0.009)	− 5.748*** (− 8.537, − 2.959)	8.169 (− 0.083, 16.420)	7.539*** (3.099, 11.980)	− 3.908* (− 7.125, − 0.691)
ECOG-PS: 2 *(Reference group* = *0)*	− 0.194*** (− 0.244, − 0.144)	− 12.381*** (− 15.524, − 9.238)	11.106 (− 1.258, 23.471)	18.784*** (13.795, 23.772)	− 11.028*** (− 14.650, − 7.405)
ECOG-PS: 3/4 *(Reference group* = *0)*	− 0.513*** (− 0.580, − 0.447)	− 24.238*** (− 28.359, − 20.117)	50.769*** (21.976, 79.563)	31.110*** (24.486, 37.734)	− 19.480*** (− 24.247, − 14.712)
Histology: squamous *(Reference group: non-squamous)*	− 0.003 (− 0.038, 0.033)	1.572 (− 0.652, 3.797)	− 0.345 (− 8.840, 8.149)	− 0.870 (− 4.416, 2.675)	0.628 (− 1.940, 3.196)
Current stage: stage IV *(Reference group: stage IIIb)*	− 0.081** (− 0.131, − 0.032)	− 1.342 (− 4.489, 1.805)	9.599 (− 1.933, 21.131)	7.070** (2.084, 12.056)	− 5.973** (− 9.576, − 2.369)
Disease duration (weeks)	− 0.001** (− 0.001, − 0.000)	− 0.067*** (− 0.090, − 0.045)	0.024 (− 0.074, 0.123)	0.067*** (0.031, 0.104)	− 0.067*** (− 0.094, − 0.041)
Current chemotherapy treatment: yes *(Reference group: no)*	0.036 (− 0.004, 0.076)	1.631 (− 0.866, 4.128)	10.977* (0.660, 21.295)	− 4.110* (− 8.105, − 0.115)	− 0.170 (− 3.047, 2.707)
Brain metastases: yes *(Reference group: no)*	0.001 (− 0.052, 0.054)	− 1.160 (− 4.475, 2.155)	6.278 (− 6.121, 18.677)	4.100 (− 1.213, 9.414)	− 2.285 (− 6.092, 1.521)
Age: 65 years or older *(Reference group*: <*65 years)*	− 0.051** (− 0.085, − 0.017)	− 4.342*** (− 6.463, − 2.220)	− 11.468 (− 27.005, 4.068)	4.212* (0.827, 7.597)	− 5.646*** (− 8.091, − 3.201)
Sex: male *(Reference group: female)*	0.024 (− 0.011, 0.059)	1.892 (− 0.298, 4.082)	− 4.922 (− 13.433, 3.588)	− 1.702 (− 5.218, 1.814)	1.909 (− 0.616, 4.435)
Smoking status: current/ex-smoker *(Reference group: never smoked)*	− 0.005 (− 0.045, 0.036)	− 5.001*** (− 7.561, − 2.441)	7.081 (− 1.166, 15.328)	7.961*** (3.849, 12.074)	− 3.968** (− 6.917, − 1.020)
Country: Germany *(Reference group: France)*	0.176*** (0.136, 0.216)	4.442*** (1.956, 6.929)	− 15.309** (− 26.217, − 4.401)	− 5.888** (− 9.889, − 1.888)	− 3.780** (− 6.647, − 0.914)
Country: Italy *(Reference group: France)*	0.257*** (0.217, 0.298)	1.003 (− 1.518, 3.524)	− 9.619 (− 20.889, 1.651)	− 10.646*** (− 14.694, − 6.597)	1.724 (− 1.173, 4.622)

	Caregiver outcomes				
	EQ-5D-3L	EQ VAS	WPAI-GH: overall work impairment	WPAI-GH: activity impairment	Zarit burden index
N	391	389	153	393	396
Adjusted R^2^	0.0695	0.1192	0.0107	0.1964	0.0828
ECOG-PS: 1 *(Reference group* = *0)*	0.022 (− 0.030, 0.075)	− 2.082 (− 6.922, 2.759)	13.566* (0.609, 26.524)	11.156** (4.159, 18.152)	5.071* (0.735, 9.408)
ECOG-PS: 2 *(Reference group* = *0)*	− 0.025 (− 0.083, 0.032)	− 3.776 (− 9.063, 1.512)	1.265 (− 13.145, 15.676)	8.846* (1.155, 16.537)	7.620** (2.869, 12.371)
ECOG-PS: 3/4 *(Reference group* = *0)*	− 0.068 (− 0.144, 0.008)	− 7.178* (− 14.167, − 0.189)	3.669 (− 14.829, 22.166)	26.375*** (16.827, 35.923)	15.662*** (9.780, 21.544)
Histology: squamous *(Reference group: non-squamous)*	0.005 (− 0.036, 0.045)	− 0.029 (− 3.706, 3.648)	− 8.511 (− 19.060, 2.037)	− 1.649 (− 6.971, 3.673)	− 1.156 (− 4.405, 2.093)
Current stage: stage IV *(Reference group: stage IIIb)*	− 0.029 (− 0.106, 0.049)	− 2.052 (− 9.084, 4.980)	6.470 (− 13.696, 26.637)	− 0.298 (− 9.558, 8.963)	2.063 (− 3.756, 7.882)
Disease duration (weeks)	0.000 (− 0.000, 0.000)	− 0.002 (− 0.040, 0.036)	0.035 (− 0.049, 0.119)	0.083** (0.026, 0.139)	0.006 (− 0.028, 0.039)
Current chemotherapy treatment: yes *(Reference group: no)*	0.017 (− 0.029, 0.062)	− 0.744 (− 4.914, 3.427)	0.184 (− 11.101, 11.470)	0.689 (− 5.328, 6.706)	− 1.793 (− 5.471, 1.885)
Brain metastases: yes *(Reference group: no)*	− 0.014 (− 0.067, 0.038)	− 1.002 (− 5.814, 3.810)	3.970 (− 9.180, 17.121)	2.842 (− 4.102, 9.786)	0.563 (− 3.783, 4.908)
Age: 65 years or older *(Reference group*: <*65 years)*	0.026 (− 0.015, 0.066)	3.040 (− 0.653, 6.733)	2.349 (− 7.327, 12.024)	− 3.772 (− 9.117, 1.573)	− 1.609 (− 4.905, 1.688)
Sex: male *(Reference group: female)*	− 0.043* (− 0.085, − 0.000)	− 1.571 (− 5.401, 2.260)	− 1.128 (− 10.435, 8.178)	− 2.203 (− 7.727, 3.321)	0.363 (− 3.044, 3.771)
Smoking status: Current/ex-smoker *(Reference group: never smoked)*	− 0.001 (− 0.049, 0.047)	− 2.976 (− 7.368, 1.416)	5.845 (− 5.750, 17.440)	8.446** (2.068, 14.823)	4.985* (1.026, 8.945)
Country: Germany *(Reference group: France)*	0.047* (0.002, 0.092)	2.243 (− 1.894, 6.380)	− 11.663* (− 22.825, − 0.501)	− 12.747*** (− 18.769, − 6.726)	− 0.740 (− 4.445, 2.965)
Country: Italy *(Reference group: France)*	0.054* (0.008, 0.101)	− 7.737*** (− 12.026, − 3.448)	4.861 (− 9.050, 18.772)	− 0.429 (− 6.559, 5.701)	0.989 (− 2.768, 4.746)
Caregiver’s age (years)	− 0.002* (− 0.003, − 0.000)	− 0.343*** (− 0.478, − 0.208)	0.033 (− 0.440, 0.506)	0.299** (0.100, 0.497)	− 0.009 (− 0.131, 0.112)
Caregiver’s sex: male *(Reference group: female)*	0.033 (− 0.009, 0.074)	0.031 (− 3.783, 3.844)	− 2.447 (− 11.933, 7.038)	− 2.849 (− 8.483, 2.786)	− 2.350 (− 5.754, 1.055)

All covariates relate to the patient unless otherwise specified

*ECOG-PS* Eastern Cooperative Oncology Group Performance Status, *EORTC QLQ-C30* European Organization for the Research and Treatment of Cancer Quality of Life Questionnaire-Core 30, *EQ-5D-3L* EuroQol 5-Dimension 3-Level, *VAS* visual analogue scale, *WPAI-GH* work productivity and activity impairment: general health, *ZBI* Zarit burden interview

* *p* < 0.05, ** *p* < 0.01, *** *p* < 0.001; data presented are coefficient (95% confidence interval)

Significantly worse outcomes were also noted for older patients (with the exception of overall work impairment), patients with a worse stage of disease (EQ-5D-3L, activity impairment and global health status), current smokers (EQ-VAS, activity impairment and global health status) and a longer length of diagnosis (with the exception of overall work impairment) (Table 4).

### Humanistic burden on caregivers

**Fig. 2:**
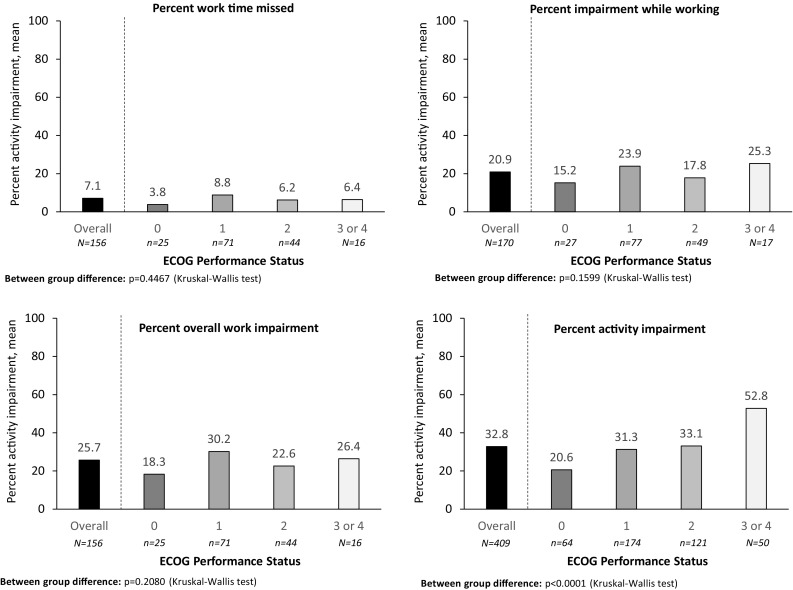
Caregiver WPAI stratified by patient ECOG Performance status

Caregivers reported a mean EQ-5D-3L utility index of 0.89 (SD 0.18) (Fig. 2). A statistically significant difference in the distribution of EQ-5D-3L utility indices across ECOG-PS subgroups was observed (*p* = 0.0072), although no clear trend between ECOG-PS and caregiver EQ-5D-3L was apparent. Anxiety/depression was the only domain that differed significantly across ECOG-PS subgroups with 34.8% of caregivers of patients with an ECOG-PS of 0 reporting some/extreme problems with anxiety/depression increasing to 66.7% of caregivers of patients with an ECOG-PS of 3 or 4 (*p* = 0.0150). When stratified by comorbid emphysema and anxiety/depression (Supplementary Table B), no differences in the distribution of EQ-5D-3L utility indices or EQ-5D VAS were observed.

**Fig. 3:**
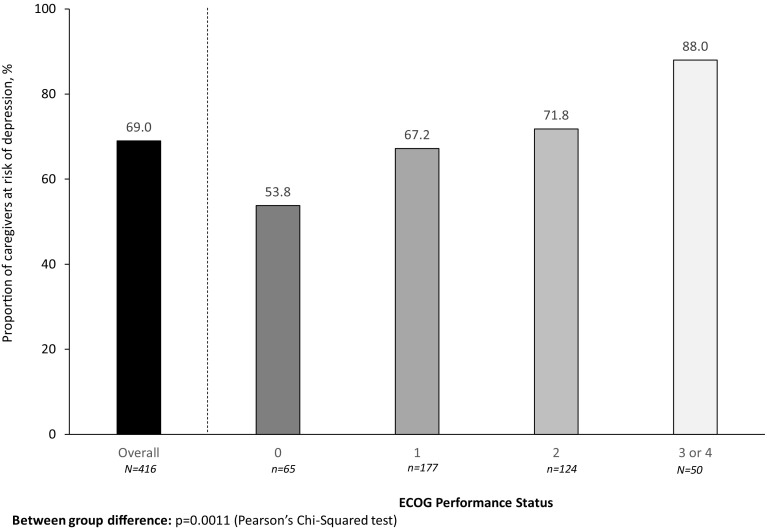
Caregiver risk of depression (ZBI) stratified by patient ECOG performance status

The distribution of activity impairment differed significantly across ECOG-PS subgroups (means: 52.8% ECOG-PS 3/4 vs 20.6% ECOG-PS 0; *p* < 0.0001), indicating a trend of greater impairment with declining functional status (Table 5; Fig. 3). This finding was also observed between caregivers caring for patients with and without comorbid anxiety/depression (means 42.8% vs 30.0%, respectively; *p* < 0.0001) (Supplementary Table B).

**Table 5:** Humanistic burden for caregivers overall and stratified patient ECOG performance status

Characteristic	Overall (*N* = 427)	Patient ECOG performance status				*P* value
		0 *n* = 66	1 *n* = 181	2 *n* = 129	3 or 4 *n* = 51	
EQ-5D-3L utility index, mean (SD)	0.89 (0.18)	0.90 (0.16)	0.92 (0.13)	0.87 (0.21)	0.82 (0.25)	0.0072^a^
EQ-5D-3L						
Mobility domain, %						
No/some/extreme problems	87.3/12.2/0.5	86.4/13.6/0	90.5/9.5/0	86.6/11.8/1.6	76.9/23.1/0	0.1367^b^
Self-care, %						
No/some/extreme problems	92.2/7.8/0	95.5/4.5/0	94.4/5.6/0	88.2/11.8/0	89.7/10.3/0	0.1442^b^
Usual activities, %						
No/some/extreme problems	89.1/10.7/0.2	90.9/9.1/0	90.5/9.5/0	87.5/11.7/0.8	84.6/15.4/0	0.6259^b^
Pain/discomfort, %						
No/some/extreme problems	78.4/21.1/0.5	80.3/19.7/0	82.1/17.3/0.6	76.6/23.4/0	64.1/33.3/2.6	0.0802^b^
Anxiety/depression, %						
No/some/extreme problems	58.8/32.7/8.5	65.2/21.2/13.6	62.8/32.2/5.0	57.8/31.3/10.9	33.3/59.0/7.7	0.0150^b^
EQ-5D VAS, mean (SD)	80.3 (16.8)	82.1 (16.6)	82.3 (14.8)	78.9 (18.6)	73.1 (17.8)	0.0186^a^
Employment status, %						0.0979^b^
Working full time	152 (36.2)	25 (39.1)	68 (37.6)	43 (34.1)	16 (32.7)	
Working part time	37 (8.8)	6 (9.4)	19 (10.5)	10 (7.9)	2 (4.1)	
Unemployed	35 (8.3)	4 (6.3)	14 (7.7)	14 (11.1)	3 (6.1)	
Student	6 (1.4)	0 (0)	2 (1.1)	4 (3.2)	0 (0)	
Homemaker	84 (20.0)	18 (28.1)	40 (22.1)	15 (11.9)	11 (22.4)	
Retired	106 (25.2)	11 (17.2)	38 (21.0)	40 (31.7)	17 (34.7)	
ZBI, mean (SD)	31.3 (15.0)	24.5 (13.3)	30.0 (14.9)	32.8 (13.8)	40.8 (15.2)	< 0.0001^a^
ZBI, n (%)						< 0.0001^b^
Little/no burden (0–20)	103 (24.8)	25 (38.5)	44 (24.9)	29 (23.4)	5 (10.0)	
Mild/moderate burden (21–40)	200 (48.1)	34 (52.3)	88 (49.7)	59 (47.6)	19 (38.0)	
Moderate/severe burden (41–60)	101 (24.3)	6 (9.2)	41 (23.2)	34 (27.4)	20 (40.0)	
Severe burden (61–88)	12 (2.9)	0	4 (2.3)	2 (1.6)	6 (12.0)	

*EORTC QLQ-C30* EORTC QoL Questionnaire, *EQ-5D-3L* EuroQol five-dimensional questionnaire three level version, *SD* standard deviation, *VAS* visual analogue scale, *WPAI-GH* work productivity and activity impairment: general health, *ZBI* Zarit burden index

^a^Kruskal–Wallis test performed

^b^Chi-squared test performed

A statistically significant difference in the distributions of ZBI was observed across ECOG-PS subgroups (*p* < 0.0001), and there was an apparent trend of increased burden with declining functionality (Table 5). A significantly higher proportion of caregivers were considered at risk of depression as patient functionality declined (53.8% [ECOG-PS 0], 67.2% [ECOG-PS 1], 71.8% [ECOG-PS 2] and 88.0% [ECOG-PS 3/4]; *p* = 0.0011). The distribution of ZBI scores also differed significantly between caregivers of patients with and without comorbid emphysema (means: 35.8 vs 30.7, respectively; *p* = 0.0323) and between caregivers of patients with and without comorbid anxiety/depression (means 36.0 vs 29.9, respectively; *p* = 0.0006).

#### Regression analyses

Regression analyses indicated that a patient’s declining functional status was associated with greater activity impairment and burden for the caregiver. An ECOG-PS score of 1, 2 or 3/4 was associated with a 11.16 (*p* < 0.01), 8.85 (*p* < 0.05) and 26.38 (*p* < 0.05) increase, respectively, in activity impairment, compared to an ECOG-PS of 0, and a 5.07 (*p* < 0.05), 7.62 (*p* < 0.01) and 15.66 (*p* < 0.001) increase, respectively in ZBI, compared to an ECOG-PS of 0 (Table 4).

Caregivers of patients who currently smoked had significantly greater activity impairment (*p* < 0.01) and ZBI scores (*p* < 0.05), whilst older caregivers also had significantly worse EQ-5D-3L utility indices (*p* < 0.05) and EQ-VAS score (*p* < 0.001) and significantly greater activity impairment (*p* < 0.01).

## Discussion

The analyses presented here show that deteriorating patient functionality (ECOG-PS), as measured by the treating physician, is accompanied by worsening outcomes related to health utility (EQ-5D-3L), activity impairment (WPAI) and reduction in QoL. Our data also highlight the increased risk of depression for caregivers of patients with aNSCLC, an observation consistent with previous studies [5, 24].

There is a paucity of data on the impact of clinical features of aNSCLC on patient and caregiver burden. Recent small studies have highlighted the impact of patient HRQoL [12], lung cancer symptoms and the presence of anxiety or depression as factors influencing caregiver burden [13]. Our analyses have extended these observations to a larger cohort of patients (*n* = 1030) and their caregivers (*n* = 427) and provided a comprehensive evaluation of the impact of patient physical functionality (ECOG-PS) and the presence of comorbid emphysema or anxiety/depression on the humanistic burden for caregivers.

The current analysis extends these observations and has confirmed that for patients with aNSCLC, QoL and, for those still employed, performance at work, is significantly impacted by functional status and that this impact increases with increasing functional disability. In this retrospective analysis, patients with poorer functioning were less likely to receive palliative systemic therapy, and yet it would be these patients who could potentially benefit the most from interventions and treatments to improve their outcomes. Although chemotherapy has been shown to improve survival for patients with aNSCLC [25], a reluctance to expose patients with poor functional status to chemotherapy and the attendant toxicities may contribute to this apparent undertreatment of patients. This is consistent with a recent report confirming patients with aNSCLC often receive little or no palliative systemic therapy [26].

We have previously shown that caregivers of patients with aNSCLC incur a significant humanistic burden in terms of their health state perception, QoL, ability to work and work performance [27]. For caregivers of patients with aNSCLC, the impact of functional status was observed mainly in terms of work-related activity impairment and perceived burden using the ZBI. We had also previously shown that caregivers of patients with aNSCLC provide an average of 29.5 h each week providing care, a figure that is close to accepted definitions of full-time occupation [28]. Employed caregivers may, therefore, incur the physical and emotional burden of two occupations with little time to focus on their own personal health and well-being. These observations suggest that interventions to maintain patients functioning, or indeed the avoidance of interventions that may markedly worsen patient functioning, have the potential to maintain patient QoL, improve caregiver work activity and reduce caregiver risk for anxiety/depression as a result of their caregiving activities. The regression analyses indicate that a patient’s declining functional status significantly impacts both the patient’s and the caregiver’s outcomes. However, it should be noted that the relatively low R-squared values for some of the models, in particular for the caregiver outcomes, indicate that the covariates included in the regression analyses do not fully explain all the variability in the outcome. Further research is warranted to identify additional covariates that may impact the patient and caregiver outcomes assessed within this study population.

Comorbid conditions may also add to the functional impairment for patients with aNSCLC. In a recent retrospective study of 6662 US-based patients with lung cancer, 51% had at least one comorbidity and 18% had four or more comorbidities [29]. This analysis also found that the type of comorbidity influenced both treatment selection and survival outcomes for patients. A database analysis conducted in Sweden also found that comorbidities contribute to a poor prognosis for patients with NSCLC [30]. Despite the apparent prevalence of comorbidities among patients with lung cancer, there is little or no data available on their impact on patient or caregiver burden. The presence of comorbid emphysema or anxiety/depression imposed further detrimental effects on patient HRQoL, activity impairment and overall burden on patients and on activity impairment and overall burden for caregivers. These observations highlight the need to take a holistic approach and to recognise and address additional health concerns, including anxiety/depression and comorbid lung conditions, in patients with aNSCLC to relieve the burden of the disease on patients and their caregivers.

The strength of this study lies in the linking of physician reported data, with that reported by patients and their caregivers. As such, this point-in-time, multi-country, multi-dimensional approach to data collection has permitted alignment of patient characteristics, with patient and caregiver burden. This linked approach, combined with a large cohort of > 1000 patients and > 400 caregivers, permitted a statistical analysis of the association between patient characteristics and patient and caregiver burden. Although not addressed directly in the current analysis, the patient–caregiver population was drawn from three EU countries, Italy, France and Germany, which are culturally very different in their attitudes/approach to caregiving including factors such as the availability of formal care, as well as the availability and affordability of dedicated palliative care teams to support both patients and their informal caregivers.

As with all point-in-time analyses, the current study provides a snapshot of the status of patients and their caregivers. In terms of limitations, while participant recall and self-reporting must be acknowledged, the effects of these were minimised in selecting validated instruments that don’t require a long recall period. When considering the generalizability of the results reported here it is important to note that the data analysed are only representative of those patients attending for physician consultation and for those caregivers who accompanied the patients to their consultations. With reference to the caregiver cohort, the demographics of the caregivers included here are consistent with those reported for other cohorts of caregivers of patients with advanced cancers, being largely older female relatives although the small number of caregiver responses when stratifying across patient ECOG-PS and comorbidities limit the conclusions that can be drawn [31, 32]. Nonetheless, the analyses presented here highlight key areas for future research including the impact of patient physical functioning and comorbidities on caregiver burden and psychological health.

The findings presented here reinforce the need to develop appropriate supportive and personalised interventions for patients and their caregivers. Approaches that can minimise or delay deterioration in patient performance status have a benefit to both patients and caregivers. This is particularly important given the central role informal caregivers play in supporting NSCLC patients. The data presented here highlight the need for interventions to maintain patients’ physical function and relieve the impact of comorbid conditions with the aim of relieving the humanistic burden both for patients and their caregivers.

## Electronic supplementary material

Below is the link to the electronic supplementary material.

Supplementary material 1 (DOCX 20 KB)
