# Supplementary material for: The humanistic burden of advanced non-small cell lung cancer (NSCLC) in Europe: a real-world survey linking patient clinical factors to patient and caregiver burden
Source: Qual Life Res. 2019 Mar 2;28(7):1849–61. doi: 10.1007/s11136-019-02152-6 (PMC6571083; doi:10.1007/s11136-019-02152-6)
Supplement: Supplementary file 1 — Supplementary material 1 (DOCX 20 KB) [file 11136_2019_2152_MOESM1_ESM.docx]

**The Humanistic Burden of Advanced Non-Small Cell Lung Cancer (NSCLC) in Europe – A Real-World Survey Linking Patient Clinical Factors to Patient and Caregiver Burden**

Wood R, Taylor-Stokes G, Smith F, Chaib C

**Supplementary material**

**Supplementary Table A:** Humanistic burden of patients overall and stratified by presence of comorbid emphysema and comorbid anxiety/depression

| **Characteristic** | **Emphysema** | | | **Anxiety/depression** | | |
| --- | --- | --- | --- | --- | --- | --- |
|  | **No**  **n=919** | **Yes**  **n=107** | **p-value** | **No**  **n=814** | **Yes**  **n=212** | **p-value** |
| EQ-5D-3L utility index, mean (SD) | 0.67 (0.31) | 0.61 (0.35) | 0.0278^a^ | 0.70 (0.30) | 0.55 (0.34) | <0.0001^a^ |
| EQ-5D-3L  Mobility domain, %  No/some/extreme problems  Self-care, %  No/some/extreme problems  Usual activities, %  No/some/extreme problems  Pain/discomfort, %  No/some/extreme problems  Anxiety/depression, %  No/some/extreme problems | 47.4/49.2/3.4  60.9/35.0/4.0  41.2/51.9/6.9  28.9/64.4/6.8  34.7/51.0/14.3 | 35.5/58.9/5.6  46.7/45.8/7.5  33.6/55.1/11.2  21.5/68.2/10.3  44.9/43.0/12.1 | 0.0149^b^  0.0033^b^  0.0679^b^  0.0593^b^  0.0585^b^ | 48.3/48.3/3.3  62.3/34.3/3.5  44.2/49.5/6.3  31.7/61.8/6.5  40.3/48.9/10.8 | 37.7/57.5/4.7  48.6/43.4/8.0  25.9/62.7/11.3  14.2/76.3/9.5  18.4/54.7/26.9 | 0.0051^b^  0.0001^b^  <0.0001^b^  <0.0001^b^  <0.0001^b^ |
| EQ-5D VAS, mean (SD) | 57.7 (18.2) | 54.2 (17.3) | 0.0671^a^ | 58.5 (18.1) | 52.9 (17.6) | <0.0001^a^ |
| WPAI, mean (SD)  % work time missed  % impairment while working  % overall work impairment  % activity impairment | 14.9 (24.2)  30.9 (22.5)  36.7 (25.9)  51.6 (28.0) | 22.8 (35.3)  36.7 (20.7)  41.7 (24.0)  62.6 (25.1) | 0.3611^a^  0.4606^a^  0.5956^a^  0.0001^a^ | 14.1 (23.3)  29.6 (22.2)  35.2 (25.6)  50.6 (28.4) | 22.7 (32.2)  41.3 (21.9)  49.2 (24.3)  61.0 (24.1) | 0.2485^a^  0.0101^a^  0.0169^a^  <0.0001^a^ |
| Employment status, %  Working full time  Working part time  Unemployed  Student  Homemaker  Retired | 143 (15.8)  62 (6.8)  93 (10.3)  1 (0.1)  72 (7.9)  535 (59.1) | 5 (4.7)  3 (2.8)  12 (11.3)  0 (0)  9 (8.5)  77 (72.6) | 0.0193^b^ | 133 (16.6)  52 (6.5)  81 (10.1)  1 (0.1)  59 (7.4)  474 (59.3) | 15 (7.1)  13 (6.1)  24 (11.3)  0 (0)  22 (10.4)  138 (65.1) | 0.0168^b^ |
| EORTC QLQ-C30 domains, mean (SD)  Global health status  Physical functioning  Role functioning  Emotional functioning  Cognitive functioning  Social functioning  Fatigue  Nausea and vomiting  Pain  Dyspnoea  Insomnia  Appetite loss  Constipation  Diarrhoea  Financial difficulties | 48.6 (19.9)  64.1 (24.9)  58.4 (28.3)  60.6 (24.7)  70.9 (25.3)  64.7 (28.2)  45.6 (25.4)  23.9 (23.5)  35.7 (25.4)  38.3 (26.8)  36.0 (28.7)  36.1 (28.6)  21.8 (24.8)  13.6 (21.1)  22.5 (26.3) | 43.6 (19.1)  58.1 (24.8)  49.1 (28.9)  59.0 (23.5)  67.6 (25.7)  61.8 (27.9)  51.8 (23.6)  24.8 (26.6)  40.0 (25.4)  42.5 (25.0)  39.9 (29.8)  39.9 (31.3)  24.8 (26.8)  12.6 (22.3)  24.3 (28.4) | 0.0089^a^  0.0079^a^  0.0012^a^  0.3495^a^  0.1619^a^  0.2583^a^  0.0070^a^  0.8578^a^  0.0945^a^  0.1191^a^  0.2136^a^  0.2530^a^  0.3144^a^  0.3800^a^  0.6696^a^ | 48.9 (20.3)  65.4 (25.0)  59.2 (28.6)  62.9 (23.9)  71.9 (24.9)  66.6 (27.6)  44.5 (25.3)  23.8 (24.1)  34.3 (25.6)  37.4 (27.0)  34.3 (28.5)  35.7 (29.1)  20.7 (24.2)  13.9 (21.5)  20.7 (25.1) | 44.8 (17.7)  56.0 (23.4)  50.7 (27.1)  51.0 (24.6)  65.4 (26.5)  55.7 (28.5)  53.0 (23.7)  24.8 (23.0)  43.3 (23.4)  43.9 (24.6)  44.4 (28.6)  39.8 (27.8)  27.8 (27.1)  12.0 (19.9)  30.3 (30.4) | 0.0023^a^  <0.0001^a^  <0.0001^a^  <0.0001^a^  0.0011^a^  <0.0001^a^  <0.0001^a^  0.3428^a^  <0.0001^a^  0.0012^a^  <0.0001^a^  0.0583^a^  0.0005^a^  0.2493^a^  <0.0001^a^ |

SD, Standard Deviation

^a^Mann-Whitney U test performed; ^b^Chi-squared test performed

**Supplementary Table B:** Humanistic burden of caregivers overall and stratified by presence of patient comorbid emphysema and comorbid anxiety/depression

| **Characteristic** | **Emphysema** | | | **Anxiety/depression** | | | |
| --- | --- | --- | --- | --- | --- | --- | --- |
|  | **No**  **n=377** | **Yes**  **n=50** | **p-value** | **No**  **n=333** | **Yes**  **n=94** | | **p-value** |
| EQ-5D-3L utility index, mean (SD) | 0.90 (0.17) | 0.85 (0.21) | 0.1207^a^ | 0.90 (0.17) | 0.87 (0.21) | | 0.1140^a^ |
| EQ-5D-3L  Mobility domain, %  No/some/extreme problems  Self-care, %  No/some/extreme problems  Usual activities, %  No/some/extreme problems  Pain/discomfort, %  No/some/extreme problems  Anxiety/depression, %  No/some/extreme problems | 88.4/11.0/0.6  93.1/6.9/0  89.5/10.2/0.3  79.9/19.6/0.6  59.1/32.4/8.5 | 79.6/20.4/0  85.7/14.3/0  85.7/14.3/0  67.3/32.7/0  57.1/34.7/8.2 | 0.0863^b^  0.0697^b^  0.4268^b^  0.0488^b^  0.8333^b^ | 87.1/12.6/0.3  92.7/7.3/0  88.3/11.7/0  79.1/20.6/0.3  61.2/30.3/8.6 | 88.4/10.5/1.2  90.6/9.4/0  91.9/7.0/1.2  75.6/23.3/1.2  50.0/41.9/8.1 | | 0.7700^b^  0.5253^b^  0.3718^b^  0.4558^b^  0.1003^b^ |
| EQ-5D VAS, mean (SD) | 80.8 (16.8) | 76.9 (17.0) | 0.0864^a^ | 81.2 (15.9) | 76.9 (19.8) | | 0.1243^a^ |
| WPAI, mean (SD)  % work time missed  % impairment while working  % overall work impairment  % activity impairment | 7.6 (11.6)  21.3 (24.9)  26.4 (27.9)  33.0 (26.0) | 2.4 (8.3)  17.1 (22.3)  20.2 (23.2)  31.3 (26.1) | 0.0175^a^  0.7276^a^  0.5411^a^  0.6585^a^ | 7.2 (12.0)  20.5 (24.1)  25.8 (27.3)  30.0 (24.9) | 6.2 (8.1)  22.5 (27.4)  25.6 (29.0)  42.8 (27.3) | | 0.8367^a^  0.8774^a^  0.6838^a^  <0.0001^a^ |
| Employment status, %  Working full time  Working part time  Unemployed  Student  Homemaker  Retired | 138 (37.2)  32 (8.6)  30 (8.1)  5 (1.3)  72 (19.4)  94 (25.3) | 14 (28.6)  5 (10.2)  5 (10.2)  1 (2.0)  12 (24.5)  12 (24.5) | 0.8544^b^ | 126 (38.5)  31 (9.5)  29 (8.9)  4 (1.2)  67 (20.5)  70 (21.4) | | 26 (28.0)  6 (6.5)  6 (6.5)  2 (2.2)  17 (18.3)  36 (38.7) | 0.0263^b^ |
| ZBI, mean (SD) | 30.7 (15.2) | 35.8 (12.9) | 0.0323^a^ | 29.9 (14.8) | 36.0 (14.6) | | 0.0006^a^ |
| ZBI, n (%)  Little/no burden (0–20)  Mild/moderate burden (21–40)  Moderate/severe burden (41–60)  Severe burden (score 61–88 | 99 (27.0)  172 (47.0)  84 (23.0)  11 (3.0) | 4 (8.0)  28 (56.0)  17 (34.0)  1 (2.0) | 0.009^b^ | 87 (26.9)  159 (49.2)  69 (21.4)  8 (2.5) | 16 (17.2)  41 (44.1)  32 (34.4)  4 (4.3) | | 0.0036^b^ |
| ZBI, At risk of depression, n (%) | 247 (67.5) | 40 (80.0) | 0.0754^b^ | 214 (66.3) | 73 (78.5) | | 0.0301^c^ |

SD, Standard Deviation

^a^Mann-Whitney U test performed; ^b^Chi-squared test performed; ^c^Fisher’s Exact test performed
